# Enhanced Cancer Metastasis in Mice Deficient in *Vasohibin-1* Gene

**DOI:** 10.1371/journal.pone.0073931

**Published:** 2013-09-16

**Authors:** Soichi Ito, Hiroki Miyashita, Yasuhiro Suzuki, Miho Kobayashi, Susumu Satomi, Yasufumi Sato

**Affiliations:** 1 Department of Vascular Biology, Institute of Development, Aging, and Cancer, Tohoku University, Sendai, Japan; 2 Department of Advanced Surgical Science and Technology, Tohoku University School of Medicine, Sendai, Japan; Cincinnati Children’s Hospital Medical Center, United States of America

## Abstract

Vasohibin-1 (VASH1) is isolated as an endogenous angiogenesis inhibitor produced by the vascular endothelium. We previously reported that tumor growth and tumor angiogenesis were augmented in *VASH1 (−/−)* mice. Here we examined whether VASH1 plays any role in cancer metastasis. When Lewis lung carcinoma (LLC) cells were inoculated in the footpad to observe spontaneous metastasis, a significant increase in lung metastasis together with inguinal lymph node metastasis was evident in the *VASH1 (−/−)* mice. Histological analyses revealed that vessels of the footpad tumor in *VASH1 (−/−)* mice were more immature, having fewer mural cells. However, when LLC cells were injected into a tail vein, the extent of lung metastasis was unchanged between wild-type mice and *VASH1 (−/−)* mice. When VASH1 in endothelial cells in culture was knocked-down by siRNA, we observed a decrease in the content of ZO-1, a component of tight junctions, which decrease resulted in increased transmigration of cancer cells across the endothelial cell monolayer. These results indicate that endogenous VASH1 tightens the endothelial barrier and makes tumor vessels resistant to cancer metastasis.

## Introduction

Cancer is the leading cause of death worldwide, and most of cancer patients die because of metastasis. The process of cancer metastasis consists of sequential steps, which include local invasion of cancer cells in the primary lesion, intravasation, traveling of cancer cell aggregates throughout the systemic circulation, arresting of cancer cell aggregates in the distant vascular beds, extravasation, and regrowth of cancer cells in the metastatic lesion [1], [2].

The tumor vasculature, a gateway of cancer cells for intravasation, is formed by the process known as angiogenesis, one of the principal hallmarks of cancers [3]. This process of angiogenesis includes the following sequential steps: detachment of surrounding mural cells from pre-existing vessels for the initiation of angiogenesis, extracellular matrix degradation by endothelial proteases, migration of endothelial cells (ECs) at the tip, proliferation of ECs at the stalk, tube formation by ECs, and redistribution and tight association of mural cells to ECs for vascular maturation and stabilization [4]. However, unlike normal blood vessels, tumor blood vessels are dilated, tortuous, and leaky due to the lack of vascular stabilization, and this abnormal architecture of tumor vessels may be responsible for the intravasation by cancer cells [5].

Angiogenesis is tightly regulated by a balance between local endogenous stimulators and inhibitors of this process [6]. A number of angiogenesis inhibitors have been identified in the mammalian body. Among them, vasohibin-1 (VASH1) is an endogenous angiogenesis inhibitor produced by the vascular endothelium having broad-spectrum anti-angiogenic activities [7], [8]. We initially isolated VASH1 as a vascular endothelial growth factor (VEGF)-inducible gene in ECs [7]. However, our subsequent analysis revealed that VASH1 is preferentially expressed in ECs of newly formed blood vessels behind the sprouting front where angiogenesis terminates [9]. Indeed, *VASH1 KO* mice contain numerous immature vessels in the area where angiogenesis should be terminated behind the sprouting front. In addition to its anti-angiogenic activity, our recent analysis further demonstrated that VASH1 increases stress tolerance of ECs and stabilizes blood vessels [10].

Histological analyses have shown that the expression of VASH1 is evident in ECs during angiogenesis under both physiological and pathological conditions including cancers [7], [11]–[21]. Thus, the expression of VASH1 can be a biomarker of angiogenesis. Alternatively, with regard to its function, we showed that increased tumor growth and tumor angiogenesis were evident when Lewis lung carcinoma (LCC) cells were inoculated into *VASH1 (−/−)* mice [12]. Indeed, decreased expression of VASH1 correlated with poor prognosis of certain human cancers [21], [22]. These observations suggest that endogenous VASH1 regulates the course of tumor angiogenesis and tumor progression. Here, we extended our analysis to the role of VASH1 in cancer metastasis. Our results indicate that VASH1 is responsible for the inhibition of cancer metastasis.

## Materials and Methods

### Cells

Lewis lung carcinoma (LLC) cells were obtained from the Cell Resource Center for Biomedical Research, Institute of Development, Aging, and Cancer, Tohoku University, and cultured in RPMI1640 medium supplemented with 10% FCS. Human umbilical vein endothelial cells (HUVECs) were obtained from Sanko Junyaku Industries (Tokyo, Japan) and cultured in type-1 collagen-coated dishes (Iwaki, Chiba, Japan) containing EBM-2 medium with growth supplements, SingleQuots, and 2% FCS (Lonza, Basel, Switzerland). LNM35 cells were described previously [8].

### Materials

The following materials were used: anti-mouse CD31 rat antibody (Fitzgerald Industries International, Concord, MA); anti-αSMA antibody (Sigma-Aldrich, St. Louis, MO); anti-LYVE-1 antibody (Acris, San Diego, CA); anti-human ZO-1 monoclonal antibody (BD Transduction Laboratories); anti-human ZO-1 polyclonal antibody (Zymed Laboratories, South San Francisco, CA); anti-VE-cadherin antibody (Santa Cruz Biotechnology, Santa Cruz, CA); Alexa fluor 488-conjugated anti-mouse IgG antibody (Molecular Probes, Eugene, OR); Alexa fluor 488-conjugated anti-rat IgG antibody (Molecular Probes); Alexa fluor 568-conjugated anti-goat IgG antibody (Molecular Probes); Alexa fluor 555-conjugated anti-mouse IgG antibody (Molecular Probes); anti-β-actin antibody (Sigma-Aldrich); HRP-conjugated anti-mouse IgG antibody (Sigma-Aldrich); HRP-conjugated anti-goat IgG antibody (Sigma-Aldrich); HRP-conjugated anti-rabbit IgG antibody (Sigma-Aldrich); Calcein-AM (Sigma-Aldrich); FITC-conjugated dextran (70 kDa, Sigma-Aldrich); 4′,6-diamidino-2-phenylindole dihydrochloride (DAPI; Molecular Probes). Anti-human VASH1 mAb (4E12) was described previously [7].

### Mouse Models of Metastasis

*VASH1(−/−)* mice of C57BL6J background were described previously [9], [12]. Wild-type (WT) C57BL6J mice (8–12 weeks; male) were obtained from Charles-River Japan (Yokohama, Japan). All animal experiments were conducted with the approval of the Tohoku University Animal Care Committee.

For the spontaneous metastasis model, 5×10^5^ LLC cells were inoculated subcutaneously in the right hind footpad of mice. Sixteen to eighteen days after inoculation, the tumor-bearing legs were resected under deep anesthesia along with the popliteal lymph nodes. Eight days after the resection, the mice were sacrificed; and their lungs were harvested, weighed, and then fixed with Tellyesniczky’s solution. Metastatic nodules on the lung surface were counted under observation with a dissecting microscope.

For the experimental metastasis model, 5×10^5^ LLC cells were injected into a tail vein. Sixteen days after the injection, the mice were sacrificed; and the lungs were then harvested and fixed with Tellyesniczky’s solution for analysis of lung metastasis.

### Immunofluorescence Analysis of Tumor Tissues

Tumor tissues were embedded in optimal cutting temperature (OCT) compound (Sakura Finetech, Tokyo, Japan) to make frozen tissue specimens, and sectioned at 6 µm. The sections were fixed with methanol for 20 minutes at −20°C, blocked for 1 hour at room temperature with PBS containing 1% BSA and 0.1% Tween-20, and stained with primary antibodies (anti-CD31 Ab, 1∶400 dilution, anti-LYVE-1 Ab, 1∶200 dilution, anti-αSMA Ab, 1∶200 dilution) at 4°C overnight, followed by Alexa 488- or Alexa 555-conjugated secondary antibodies (1∶400 dilution) and DAPI for 1 hour at room temperature. The sections were covered with fluorescent mounting medium (DAKO, Tokyo, Japan), and images were acquired with a fluorescent microscope (BZ-9000, Keyence) and analyzed with a BZ-II software (Keyence, Osaka, Japan).

### Isolation of ECs from Lungs and LLC Tumors

ECs were isolated from the tissue by the use of Magnetic Cell Sorting System (MACS, Miltenyi Biotec, Auburn, CA). Lung of WT mice and tumor tissues of WT and *VASH1 (−/−)* mice were minced and digested with collagenase II (Worthington, Freehold, NJ). For lung tissues, blood cells were removed by PBS perfusion before excision. For tumor tissues, a single sucrose step-gradient centrifugation with Histopaque 1077 (Sigma-Aldrich) was done after the digestion to remove blood cells. The cell suspensions were filtered with 40 µm cell strainer (BD). The digested tissues were processed with ACK Lysing Buffer (GIBCO), and CD31 positive ECs were isolated by MACS with CD31 antibody (Fitzgerald Industries International, Concord, MA).

### Real-time RT-PCR

Total RNA was extracted from the isolated endothelial cells by RNeasy (Qiagen), and reverse transcription was performed. Real-time PCR analysis was done using CFX96 (BIO-RAD) and SYBR premix Ex Taq (TaKaRa BIO, Otsu, Japan). The sense and antisense primer pairs were: mouse VASH1, 5′-TCAGCACAGAGAGATGAGGA-3′ and 5′-TACTGCAGCTCCCTGATGTA-3′; mouse CD31, 5′-TTCAGCGAGATCCTGAGGGTC-3′ and 5′-CGCTTGGGTGTCATTCACGAC-3′, respectively.

### Gene Silencing by Stealth siRNA

HUVECs were transfected with synthetic siRNAs in Lipofectamine RNAi max reagent (Invitrogen) according to the manufacturer’s protocol. At 12 hours after transfection, the cell culture medium was replaced with growth medium, and the cells were incubated for an additional 12 hours. Then, the cells were used for morphological analysis or harvested for experiments. Specific gene silencing was verified by Western blot analysis. The nucleotide sequences of stealth siRNAs for human VASH1 and its control were 5′-CAA GGA CCG GAA GAA GGA UGU UUC U-3′ and 5′-CAA CCA AGG AGA GGA GUA UUG GUC U-3′, respectively.

For the rescue experiment, HUVECs were transfected with siRNAs for 3′ un-translated region (UTR) of human VASH1 and the expression vector of human VASH1 cDNA lacking 3′ UTR. At 48 hours after transfection, the cells were extracted for Western blotting. The nucleotide sequences of stealth siRNAs for 3′ UTR of human VASH1 and its control were 5′-ATA AGA TGC ACC CAA CTC CCA GAG A-3′ and 5′-ATA GAA AGG TAC CCA CCA CTC CAG A-3′, respectively.

### Western Blot Analysis

Western blot analysis was performed as previously described [7]. Briefly, HUVECs treated with siRNA were lysed with RIPA buffer (Nacalai Tesque, Tokyo, Japan); and the lysates were then separated by SDS-PAGE on a 7.5% separating gel and subsequently transferred to nitrocellulose membranes. The membranes were blocked for 1 hour at room temperature with PBS containing 1% skim milk and 0.1% Tween-20, and then incubated tor 1 hour at room temperature with primary antibodies (anti-ZO-1 Ab, 1∶200 dilution; anti-VE-cadherin Ab, 1∶200 dilution; anti-β-actin Ab, 1∶10000 dilution; anti-human VASH1 antibody (4E12), 1∶500 dilution). Then, the membranes were incubated with HRP-conjugated secondary antibodies for 1 hour at room temperature, after which the blots were detected with Immobilon reagent (Millipore, Concord, MA). Images were obtained by using an LAS-4000 luminescent image analyzer (Fuji Film, Tokyo, Japan).

### Immunofluorescence of HUVECs

After siRNA treatment, HUVECs were plated on Type-1 collagen-coated glass coverslips and incubated for 48 hours. Then, the cells were fixed with 4% paraformaldehyde for 10 minutes, permeabilized with 0.2% Triton X-100 (Sigma-Aldrich) for 15 minutes, and blocked for 1 hour at room temperature with PBS containing 1% BSA and 0.1% Tween-20 (Sigma-Aldrich). They were next incubated overnight at 4°C with primary antibodies (anti-ZO-1 Ab, 1∶200 dilution; anti-VE-cadherin Ab, 1∶200 dilution). After 2 washes with PBS, the cells were incubated for 1 hour at room temperature with Alexa 488- or 568-conjugated secondary antibodies (1∶400 dilution). The glass coverslips were finally covered with fluorescent mounting medium, and images were acquired by using a fluorescence microscope (DMI6000B, Leica).

### Permeability of the Endothelial Monolayer

siRNA-treated HUVECs (1×10^5^ cells) were plated on the floor of the upper chamber of Type-1 collagen-coated Transwell permeable supports (0.4-µm pores, 24 wells; Corning) to prepare HUVEC monolayers. After incubation for 24 hours, FITC-dextran (70 kDa; 1 mg/ml in 100 µl of EBM-2 medium) was added to the upper chamber; and the FITC-dextran that had passed through the monolayer into the lower chamber was quantified by fluorometry (absorption/emission at 485/538 nm).

### Transendothelial Migration of Cancer Cells

siRNA-treated HUVECs (5×10^4^ cells) were plated on the floor of the upper chamber of Type-1 collagen-coated Transwell permeable supports (8-µm pores, 24 wells; Corning) and incubated for 24 hours to prepare monolayers of HUVECs. Then, LNM35 human lung cancer cells stained with calcein were added to the upper chambers (1×10^4^ cells/100 µl of EBM-2 medium, 0% FCS), with the lower chambers containing 600 µl of RPMI1640 medium with 10% FCS. Six hours later, the number of cells that had transmigrated to the lower side of the membranes was counted under observation with a fluorescence microscope.

### Statistical Analysis

The statistical significance of differences between groups was evaluated by use of the unpaired ANOVA, and *P* values were calculated by performing the unpaired Student’s *t* test.

## Results

### Increase in Spontaneous Metastasis in *VASH1 (−/−)* Mice

**Figure 1:**
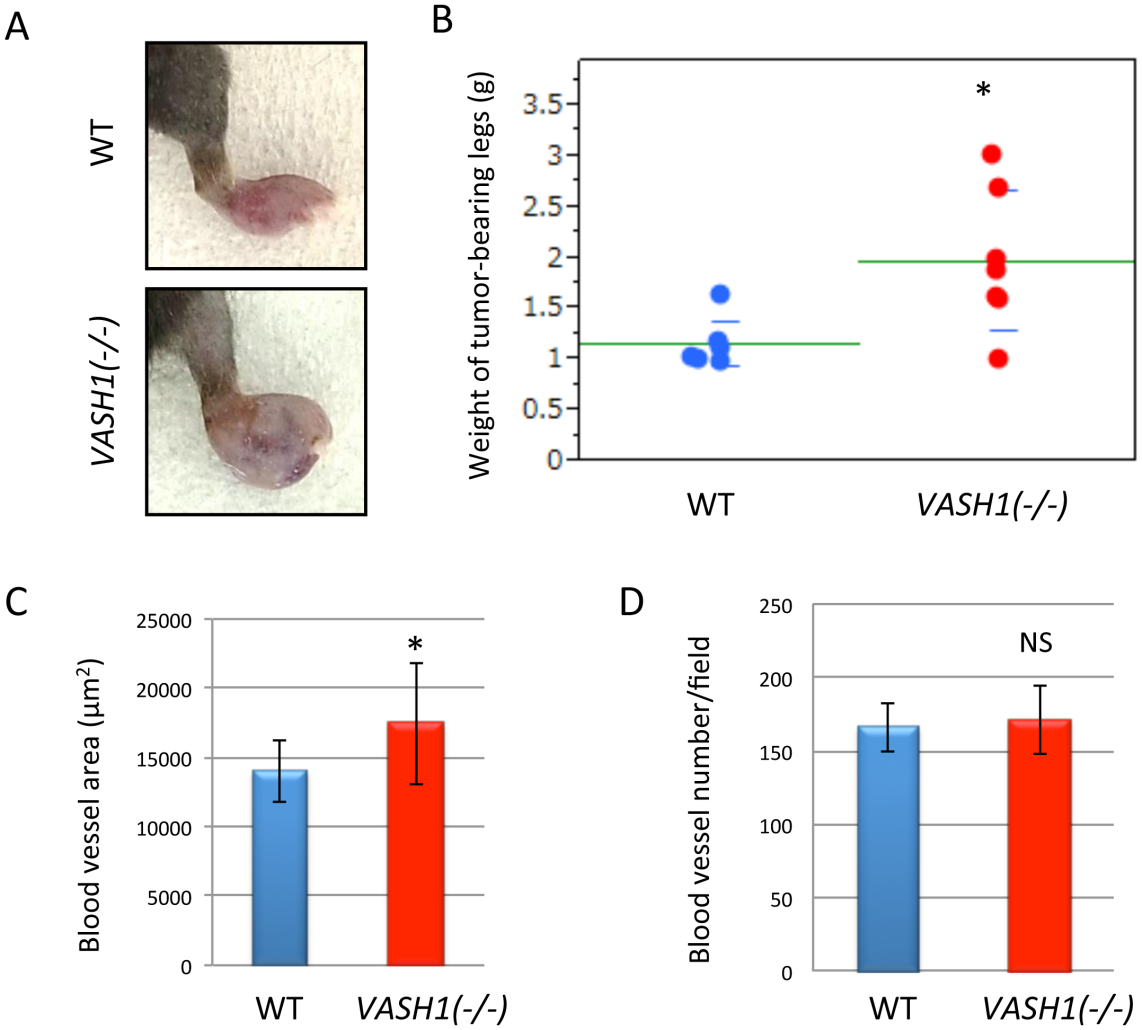
Primary tumors in the footpad. LLC cells were inoculated in the right hind footpad of WT and *VASH1 (−/−)* mice. A: Gross appearance on day 16 to 18 after the inoculation. B: Weight of tumor-bearing legs. *p<0.05. C: Tumor sections were immunostained with CD31, and the vascular area was determined as described in Materials and Methods. Means and SDs are shown (N = 7). *p<0.05. D: Tumor sections were immunostained with CD31, and vascular number was determined as described in Materials and Methods. Means and SDs are given (N = 7). NS: not significant.

**Figure 2:**
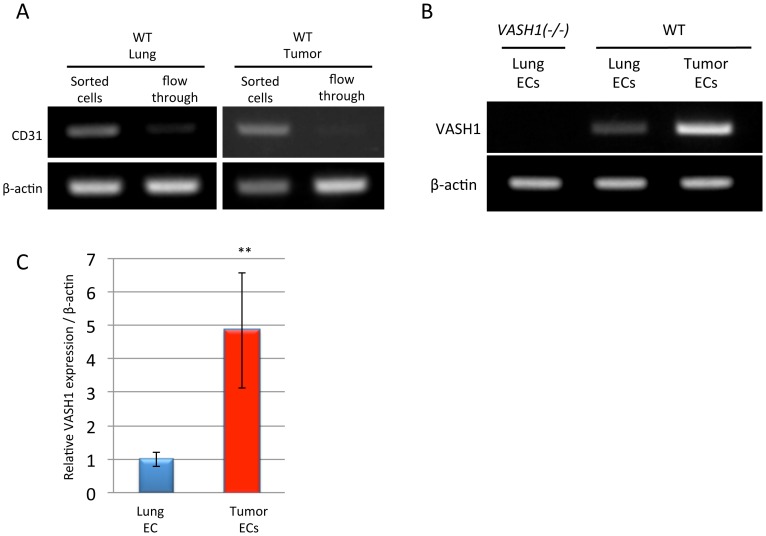
Expression of VASH1 in ECs. A: The expression of CD31 expression in magnetically sorted cells from normal lungs or tumor tissues and flow through fraction was analyzed by RT-PCR. B: The expression of mouse VASH1 in lungs from *VASH1(−/−)* mice, and in normal lungs and tumor tissues from WT mice were determined by RT-PCR. C: The expression of mouse VASH1 expression in normal lungs and tumor tissues from WT mice was quantitated by real-time RT-PCR and compared (N = 3). **p<0.01.

To evaluate the possible role of VASH1 in cancer metastasis, we inoculated LLC cells subcutaneously in the right hind footpad of WT and *VASH1 (−/−)* mice. Sixteen to eighteen days after the inoculation, the tumor-bearing legs were resected; and the size and weight of the tumors were determined. Tumors in *VASH1 (−/−)* mice grew bigger (Fig. 1A and B). Tumors in the *VASH1 (−/−)* mice showed an increased vascular area (Fig. 1 C and D). We isolated normal ECs from WT mice or tumor associated ECs from WT and *VASH1 (−/−)* mice, and compared the expression of VASH1. The expression of VASH1 in ECs of tumor vessels was significantly higher than that in those of normal quiescent vessels in WT mice, while the expression of VASH1 was absent in *VASH1 (−/−)* mice (Figure 2).

**Figure 3:**
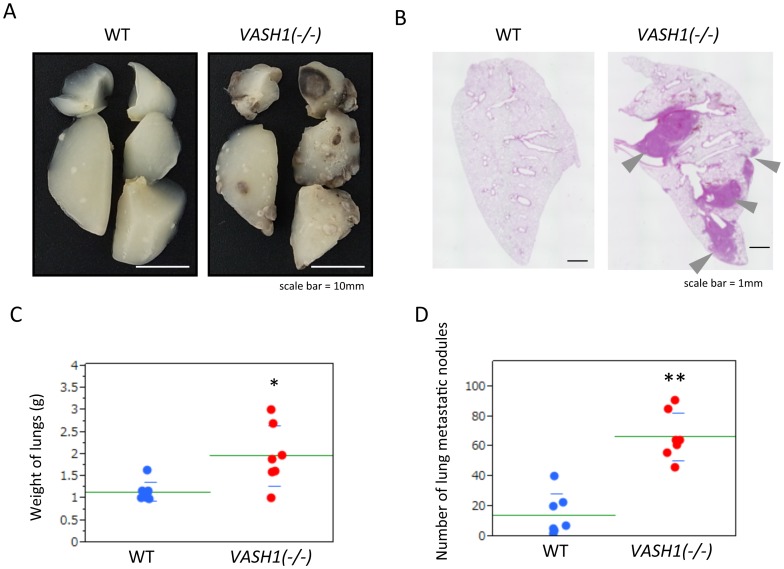
Lung metastasis in the spontaneous metastasis model. Eight days after leg resection, mice were sacrificed, and lung metastasis was evaluated. A: Gross appearance. B: Hematoxylin and eosin (H&E) staining. Arrowheads indicate metastasis. Bar = 1 mm. C: Weight of lungs from WT and *VASH1 (−/−)* mice is shown (N = 7). *p<0.05. D: Number of metastatic nodules in the lungs of WT and *VASH1 (−/−)* mice is given (N = 7). **p<0.01.

Eight days after resection of the tumor-bearing legs, we sacrificed the mice and determined the extent of lung metastasis. Gross appearance and sections of lungs revealed a significant increase in the number of metastatic tumors in the *VASH1 (−/−)* mice (Fig. 3A and B). This increase in metastasis in the *VASH1 (−/−)* mice was further quantified by weighing the lungs and counting the number of metastatic nodules in them (Fig. 3C and D).

The increased size of primary tumor can be one reason for the augmented metastasis in *VASH1 (−/−)* mice (Fig. 1B). However, the weight of tumor-bearing leg was less than 2 fold (Fig. 1B), whereas the number of metastatic nodule was more than 3 fold in *VASH1 (−/−)* mice (Fig. 3D). This proportional increase of lung metastatic nodules may indicate the acceleration of metastatic processes itself in *VASH1 (−/−)* mice. Indeed, we confirmed the significant increase of lung metastatic nodules in *VASH1 (−/−)* mice with the same weight of tumor-bearing legs (Figure S1).

**Figure 4:**
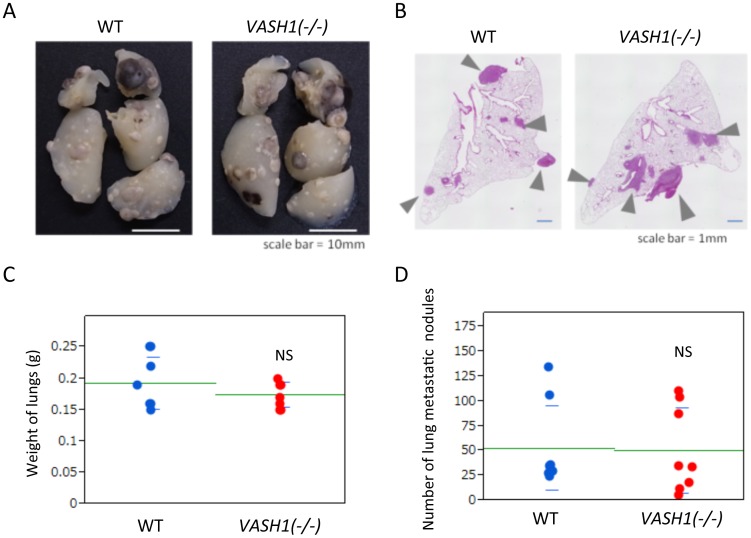
Lung metastasis in the experimental metastasis model. Eight days after injection of LLC cells via a tail vein, mice were sacrificed; and lung metastasis was then evaluated. A: Gross appearance. B: H&E staining. Arrowheads indicate metastasis. Bar = 1 mm. C: Weight of lungs from WT and *VASH1 (−/−)* mice is given (N = 8). NS: not significant. D: Number of metastatic nodules in the lungs from WT and *VASH1 (−/−)* mice is shown (N = 8). NS: not significant.

We next applied another mouse model of metastasis by injecting LLC cells directly via a tail vein. This maneuver, so-called the experimental metastasis model, skipped the initial intravasation step. What we observed was that the extent of lung metastasis was unchanged between WT and *VASH1 (−/−)* mice in this model (Fig. 4A, B, C and D).

Collectively, these results suggest that cancer metastasis was increased *VASH1 (−/−)* mice, and the intravasation by cancer cells in the primary lesion might be responsible for this increase in metastasis.

### Increase in Lymph Node Metastasis in *VASH1 (−/−)* Mice

**Figure 5:**
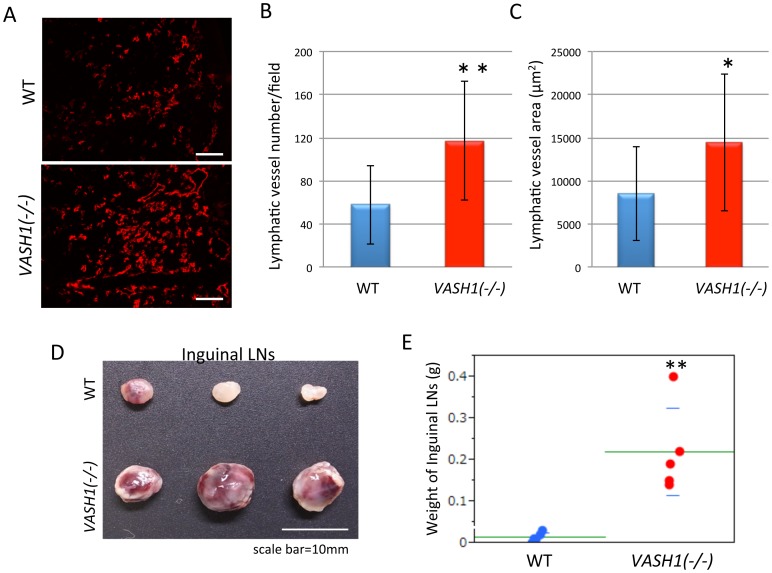
Lymphangiogenesis and LN metastasis in the spontaneous metastasis model. A: Tumor lymphatic vessels were stained with LYVE-1. Bar = 100 µm. B: Lymphatic vessel number per field in the tumor is shown (N = 4). **p<0.01. C: Lymphatic vessel area in the tumor is shown (N = 4). *p<0.05. D: The right-side inguinal LNs were recovered and photographed. Bar = 10 mm. E: Weight of the inguinal LNs is shown (N = 5). **p<0.01.

We also examined the extent of lymphangiogenesis in the footpad tumors (Fig. 5A), and observed the significant increase in number and area of lymphatic vessels in *VASH1 (−/−)* mice (Fig. 5B and C). We then evaluated the regional inguinal LN metastasis 8 days after resection of the tumor-bearing legs, and found that LN metastasis was enhanced in *VASH1 (−/−)* mice (Fig. 5D and E).

### Immature Tumor Vessels in *VASH1 (−/−)* Mice

**Figure 6:**
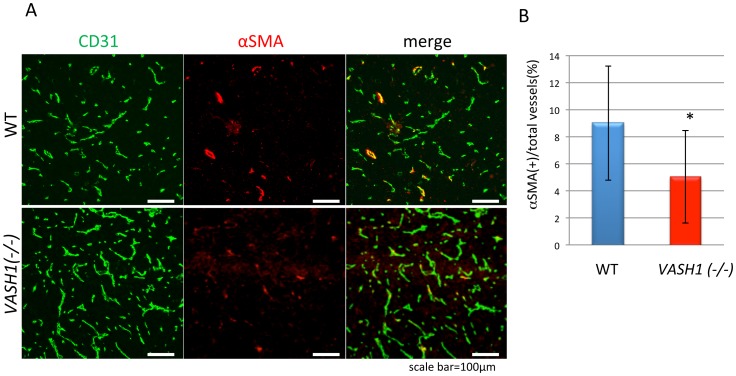
Composition of tumor vessels with respect to ECs and mural cells. A: Tumor sections from the legs of WT and *VASH1 (−/−)* mice were immunostained for CD31 and αSMA. Bar = 100 µm. B: Ratio of αSMA-positive vessels to total vessels is shown (N = 7). *p<0.05.

Normal blood vessels are mature surrounded by mural cells, but tumor blood vessels are generally immature, being deficient in mural cell coverage. Here we examined the cellular composition of the tumor vessels by immunostaining them with antibody against CD31, a marker of ECs, and antibody against αSMA, a marker of mural cells. As expected, many of the tumor vessels were not covered by mural cells in WT mice (Fig. 6A; upper panels). However, the extent of coverage by mural cells was significantly less in tumor vessels of the *VASH1 (−/−)* mice (Fig. 6A; lower panels and B).

This observation corresponds very well to our previous one [12], and this difference in mural cell coverage might be one reason for the increase in cancer cell intravasation in these *VASH1 (−/−)* mice. Nevertheless, since tumor vessels in WT mice were considerably immature, there might be an additional reason for the enhanced metastasis in *VASH1 (−/−)* mice.

### Deficient Formation of Tight Junctions in Endothelial Monolayers Lacking VASH1

**Figure 7:**
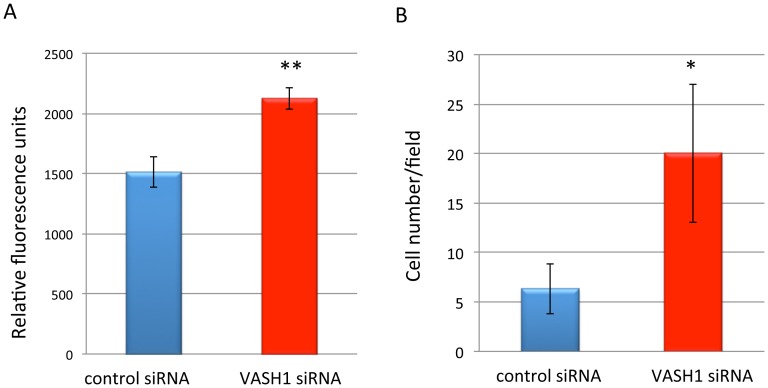
Permeability and transmigration of cancer cells across the endothelial monolayer. A: The amount of FITC-dextran that passed through monolayers of HUVECs treated with control siRNA or VASH1 siRNA was compared (N = 3). **p<0.01. B: The number of cancer cells that transmigrated through the monolayer of HUVECs treated with control siRNA or VASH1 siRNA was compared. (N = 3). *p<0.05.

We therefore examined whether there were any changes in the property of the ECs themselves when they lacked VASH1 expression. We knocked-down the expression of VASH1 in HUVECs by the use of siRNA, and then examined the permeability across the endothelial monolayer. There was a significant increase in the permeability across the monolayer of HUVECs deficient in VASH1 (Fig. 7A). We further examined the transmigration of cancer cells across the endothelial monolayer. There was a significant increase in the transmigration of cancer cells across the monolayer of VASH1-deficient HUVECs (Fig. 7B).

**Figure 8:**
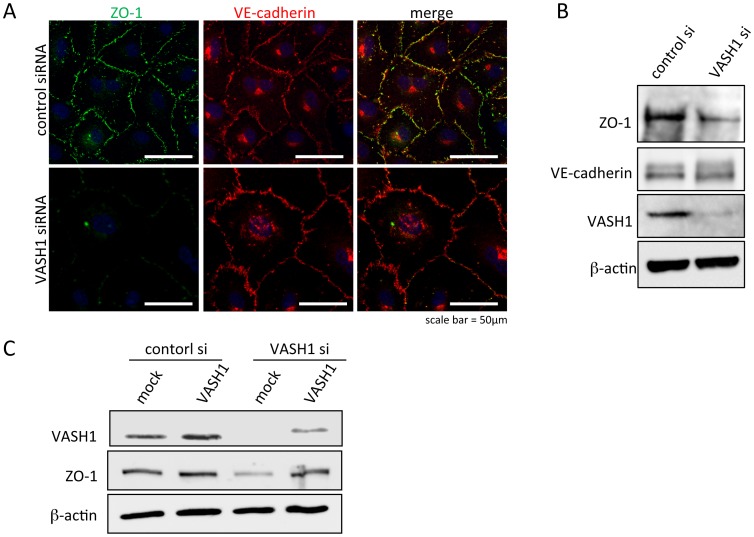
Cell-cell junctions of HUVECs. Seventy-two hours after the siRNA treatment, immunostaining for ZO-1 and VE-cadherin, components of tight junctions and adherence junctions, respectively, in HUVECs treated with control siRNA or VASH1 siRNA was performed. Bar = 50 µm. B: Protein content of ZO-1, VE-cadherin, VASH1, and β-actin in HUVECs treated with control siRNA or VASH1 siRNA was analyzed by Western blotting. C: HUVECs were transfected with the VASH1 expression vector, and stealth siRNA. The cells were extracted 48 hours later, and Western blot analysis was performed.

A factor that determines the permeability of and transmigration of cancer cells across the endothelial monolayer would be the formation of cell-cell junctions. Therefore, we examined the components of cell-cell junctions by immunostaining with antibody against ZO-1 as a marker of tight junctions and antibody against VE-cadherin as a marker of adherence junctions. There was a decrease in ZO-1, but not VE-cadherin, positive immunostaining in the monolayer of HUVECs deficient in VASH1 (Fig. 8A). This change in ZO-1 content was further confirmed by Western blotting (Fig. 8B). We further used siRNAs for 3′ UTR of human VASH1 and the expression vector of human VASH1 cDNA lacking 3′ UTR for the rescue experiment. Accordingly, siRNA should knockdown endogenous VASH1 expression but would not interfere the expression of exogenous VASH1 by the expression vector. As shown in Fig. 8C, the knocked-down of VASH1 by this siRNA also reduced the expression of ZO-1, and the co-transfection of VASH1 cDNA lacking 3′ UTR restored the expression of VASH1 and prevented the decreased of ZO-1 expression.

## Discussion

Here we examined the role of endogenous VASH1 in cancer metastasis in relation to the interaction between cancer cells and the vasculature. We employed 2 mouse models of cancer metastasis, one that evaluated the whole processes of metastasis as the spontaneous metastasis model and the other, the process of extravasation and subsequent events as the tail vein injection model. Our present analyses revealed the enhanced metastasis in *VASH1 (−/−)* mice in the spontaneous metastasis model but not in the tail vein injection model. In the spontaneous metastasis model, cancer cells transmigrate through disorganized newly formed tumor vessels. However, when cancer cells were intravenously injected in the experimental metastasis model, the process of intravasation was skipped. This might be one reason that we could not find any difference in the experimental metastasis model. We speculated that endogenous VASH1 took part in the defense against cancer cell intravasation in the primary lesion. We also propose that the tightness of endothelial barrier is more important for cancer cells to leave from the primary site.

We observed the increase of tumor lymphangiogenesis and regional LN metastasis in *VASH1 (−/−)* mice. We previously demonstrated that exogenous VASH1 inhibited tumor lymphangiogenesis and LN metastasis [8]. Importantly, our present results further explored that endogenous VASH1 possesses the inhibitory effect on tumor angiogenesis and LN metastasis. As VASH1 is preferentially produced by vascular ECs [12], this inhibitory effect of VASH1 on lymphangiogenesis is mainly mediated via a paracrine manner.

Peripheral lymphatic vessels normally lack mural cells. Thus, the increase of lymphatic vessels may directly contribute to intravasation of cancer cells to lymphatic vessels and LN metastasis. However, blood vessels are structurally different. We therefore examined the cellular composition of tumor blood vessels, and reaffirmed our previous observation that tumor blood vessels in *VASH1 (−/−)* mice are more immature, having fewer mural cells [12]. It was previously documented that deficient coverage of tumor blood vessels by mural cells is accompanied by an increased incidence of metastasis of human colorectal carcinoma cells [23]. The importance of mural cell coverage for the protection of tumor blood vessels from cancer cell intravasation was further confirmed by a series of experiments with several animal models [24]–[27]. Thus, we considered that the immature tumor blood vessels in *VASH1 (−/−)* mice might be one reason for the augmentation of cancer metastasis. So far, we could not detect any effects of VASH1 on mural cells [7]. Thus, the mechanisms governing the VASH1-mediated maintenance of blood vessel maturity remain to be elucidated.

As VASH1 is synthesized by and affects ECs by acting as an autocrine factor [7], [10], VASH1 might modify the property of the ECs themselves besides interacting with mural cells. Indeed, the knockdown of VASH1 enhanced the permeability and transmigration of cancer cells across the homotypic endothelial monolayer. These results promoted us to examine homotypic cell-cell adhesion in the lining endothelium. We indeed found that the knockdown of VASH1 in ECs resulted in a decreased content of ZO-1, a component of tight junctions, but had no effect on VE-cadherin, a component of adherence junctions. Moreover, exogenously transfected VASH1 could restore the decrease of ZO-1 induced by VASH1 siRNA.

The tight junction is the most topical structure that governs the permeability of epithelial and endothelial layers. Tight junctions in the endothelial layer function as a barrier through which liquid, molecules, and cells cannot pass [28], [29]. Thus, the tight junction should be a barrier that cancer cells must overcome for their intravasation. Indeed, the decreased tight junction in tumor vessels is accompanied by the acquisition of the metastatic phenotype in melanoma [30], and a poor prognosis in hepatocellular carcinoma [31]. Therefore, we consider that the decreased “tightness” of the tight junction might be another reason for the augmentation of cancer metastasis. The mechanism how VASH1 regulates endothelial tight junction formation remains to be elucidated.

In summary, we concluded endogenous VASH1 to be required for the resistance of the endothelium against the intravasation of cancer cells needed for metastasis. VASH1 may function to ensure tight binding between endothelial cells and the association of the endothelium with mural cells for vascular maturation, and these phenotypes resemble those of “phalanx endothelial cells” [32], [33]. Although the precise mechanisms by which VASH1 works remain to be elucidated, these functions of VASH1 can be applied to the inhibition of cancer metastasis.

## Supporting Information

Figure S1 Sixteen to eighteen days after the inoculation of cancer cells, tumor-bearing legs were resected.
(DOCX)
