# Supplementary material for: Enhanced Cancer Metastasis in Mice Deficient in Vasohibin-1 Gene
Source: PLoS One. 2013 Sep 16;8(9):e73931. doi: 10.1371/journal.pone.0073931 (PMC3774736; doi:10.1371/journal.pone.0073931)
Supplement: Figure S1 — Sixteen to eighteen days after the inoculation of cancer cells, tumor-bearing legs were resected. (DOCX) [file pone.0073931.s001.docx]

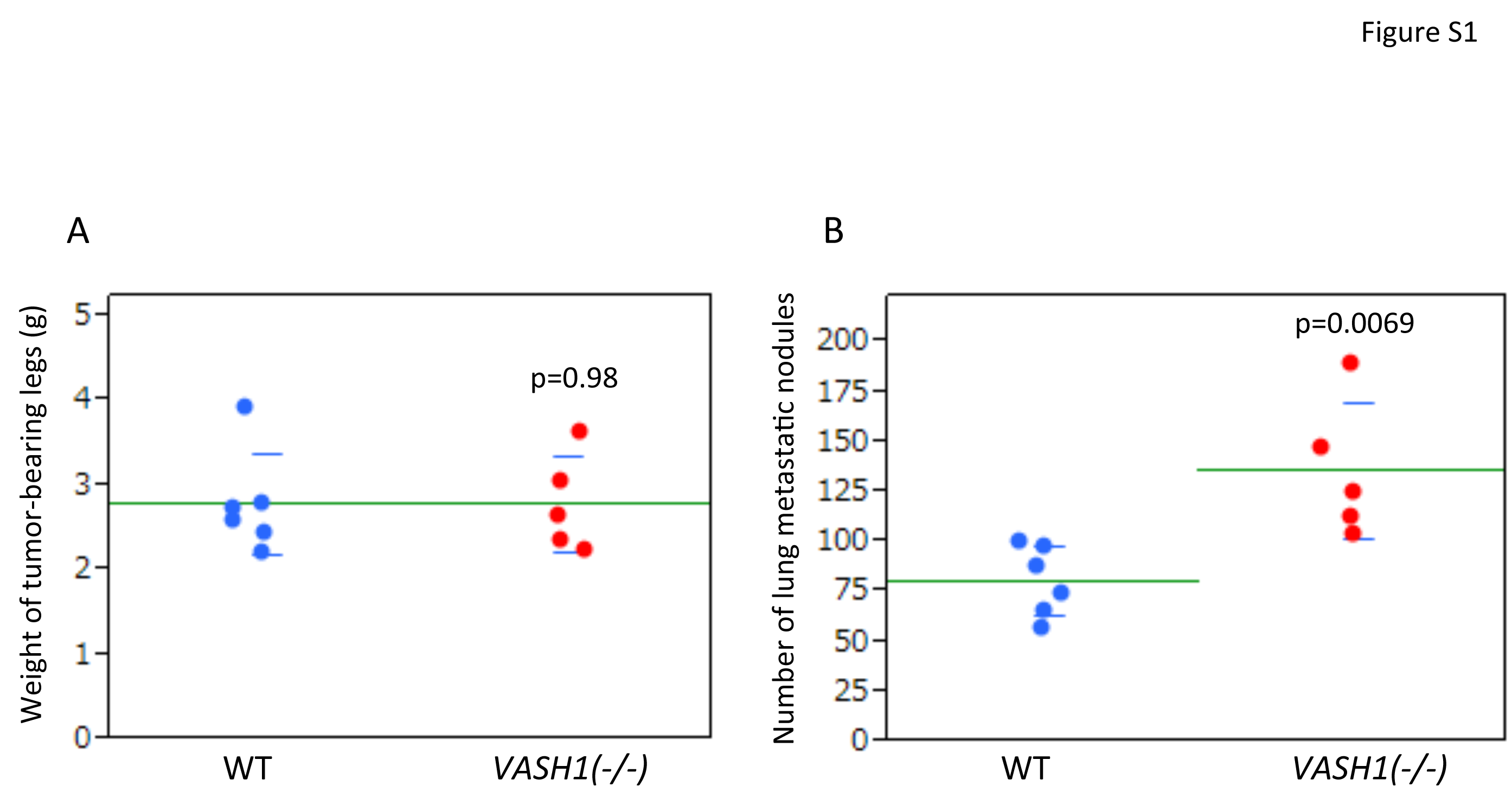


Legend for Figure S1:

Sixteen to eighteen days after the inoculation of cancer cells, tumor-bearing legs were resected. Mice with the leg weight of 2 to 4 g were further analyzed the spontaneous lung metastasis. A: Weight of legs from WT (N=6) and *VASH1 (-/-)* mice (N=5) is shown. B: Number of metastatic nodules in the lungs of WT (N=6) and *VASH1 (-/-)* mice (N=5) is given.
